# Prevalence and Determinants of Metabolic Syndrome among Women in Chinese Rural Areas

**DOI:** 10.1371/journal.pone.0036936

**Published:** 2012-05-10

**Authors:** Hui Cai, Jianping Huang, Guangfei Xu, Zili Yang, Ming Liu, Yaoping Mi, Weisheng Liu, Hongjun Wang, Derong Qian

**Affiliations:** 1 Department of Epidemiology and Medical Statistics, Nantong University, Nantong, Jiangsu, China; 2 Department of Chronic Disease and Prevention, Center for Disease Control and Prevention of Nantong, Nantong, Jiangsu, China; 3 Department of Chronic Disease and Prevention, Center for Disease Control and Prevention of Tongzhou, Nantong, Jiangsu, China; Fundación para la Prevención y el Control de las Enfermedades Crónicas No Transmisibles en América Latina (FunPRECAL), Argentina

## Abstract

**Background and Aims:**

Metabolic syndrome (MS) is prevalent in recent years but few data is reported in the rural areas in China. The aim of this study was to examine MS prevalence and its risk factors among women in rural China.

**Methods and Results:**

The Nantong Metabolic Syndrome Study (NMSS), a population based cross-sectional study, was conducted during 2007–2008 in Nantong, China. In person interviews, blood glucose and lipid measurements were completed for 13,505 female participants aged 18–74 years. The International Diabetes Federation (IDF), the US Third Report of the National Cholesterol Education Program, the Adult Treatment Panel (ATPIII) and modified ATPIII for Asian population has determined three criteria of MS. These criteria for MS were used and compared in this study. The prevalence of MS was 22.0%, 16.9% and 23.3% according to IDF, ATPIII and ATPIII-modified criteria, respectively. Levels of agreement of these criteria for MS were above 0.75. We found that vigorous-intensity of occupational physical activity was associated with a low prevalence of MS with OR of 0.76 (95% confidence interval (CI): 0.63–0.91). Rice wine drinkers (alcohol >12.8 g/day) had about 34% low risks of developing MS with OR of 0.66 (95% CI: 0.48–0.91), compared with non-drinkers. Odds ratio of MS was 1.81 (95% CI: 1.15–2.84) in women who smoked more than 20 pack-years, compared to non-smokers. Odds ratio of MS was 1.56 (95% CI: 1.25–1.95) in women who had familial history of diseases, including hypertension, diabetes and stroke, compared to women without familial history of those diseases.

**Conclusion:**

MS is highly prevalent among women in rural China. Both physical activity and rice wine consumption play a protective role, while family history and smoking are risk factors in MS development. Educational programs should be established for promoting healthy lifestyles and appropriate interventions in rural China.

## Introduction

Metabolic Syndrome (MS) is characterized by a cluster of metabolic disorders, including abdominal obesity, hyperglycemia, dyslipidemia and high blood pressure [1]. The prevalence of MS has varied markedly among different studies because of the lack of internationally agreed criteria. Different lifestyles, environmental and genetic factors across countries and populations may have effects on the prevalence of MS. The main definitions of MS have been proposed by the World Health Organization in 1999 [2], the US Third Report of the National Cholesterol Education Program, Adult Treatment Panel (ATPIII) in 2001 [3], and the International Diabetes Federation in 2005 (IDF) [4]. A revised ATPIII criterion has been proposed using a cut point for central obesity that is ethnic specific and a lower cut point for glucose intolerance (ATPIII-modified) [5] because people found that South and East Asians might develop increased visceral adiposity even with a low body mass index and waist circumference [6].

Recent reports suggest that MS criteria are met by approximately 1 in 3 adults [7], [8]. MS not only increases cardiovascular morbidity and mortality but also increases the risk of developing diabetes because its components represent major risk factors for impaired glucose metabolism [9]. Major efforts are under way to detect, treat and prevent MS as a means of lowering the risk of diabetes and cardiovascular disease (CVD) in the world [5], [10], [11]. Some behaviors and lifestyles, including physical activity, cigarette smoking and alcohol consumption, are considered to be associated with MS in some studies, but the conclusions are inconsistent in other studies [12], [13], [14], [15], [16]. Several studies conducted in China recently have evaluated the prevalence of MS and related risk factors among elderly Chinese people [17] and minors of the Chinese population [18]. Few studies focused on association of the prevalence of MS and its determinants in the rural areas in China.

In 2007, we launched the Nantong Metabolic Syndrome Study (NMSS), a population-based cross-sectional study of 20,502 participants (6,997 men and 13,505 women) aged 18–74 years in the rural area of Nantong, China. The NMSS focuses on the prevalence of MS and its determinants in both men and women in rural China because people living in rural areas have low education levels, low income, heavy labor and different behaviors and lifestyles, compared with people living in urban areas. The proportion of female drinkers, for example, is 11 percent in our study and only 3 percent or less among women living in urban China. Therefore, our study may have more statistical power to detect associations between drinking and MS than those studies conducted in urban China. The characteristics of people in our study may lead to different relations between conventional factors and the prevalence of MS and our findings will benefit people living in rural China with MS prevention. In this report we estimated the prevalence of MS in Chinese women living in rural areas according to three criteria and evaluated associations of familial history, smoking status, alcohol consumption and physical activity with the prevalence of MS. To the best of our knowledge this is the largest study for MS among women in rural China.

## Methods

### Study population

The recruitment for the NMSS started in July 2007 and completed in August 2008. A total of 24,519 residents in two townships of rural Nantong between ages of 18 to 74 years were invited to participate by trained interviewers through in-person contact. A total of 20,502 participants, including 6,997 men and 13,505 women, were enrolled in the study with a response rate of 83.6%. Reasons for non-participation are refusal (3.21%), out of area during enrollment (7.21%), and other miscellaneous reasons including poor health or hearing problems (5.98%). In our study, a rural area is defined as an area with a primary administrative unit named ‘village’. Most participants (99.5%) in our study lived in one of those villages at the time of interview and 9,323 women (69%) are farmers. The study protocols were approved by both Boards of Scientific Research of Nantong University and Nantong CDC and all participants provided written informed consent. Information was collected on socio-economic factors, dietary intake, physical activity at work and leisure time, alcohol consumption, smoking status, personally medical history and familial history. Participants were measured for height, weight and waist circumferences according to standard protocol.

The level of physical activity during leisure time (LPA) was self-reported as: no LPA (watching TV, reading and writing), light LPA (Qi Gong and some stretching exercises), moderate LPA (jogging and dancing) and vigorous LPA (playing basketball, badminton). Occupational physical activity (OPA) was divided into five groups: No job, sedentary work (typists, computer operators), light OPA (clerk, teacher), moderate OPA (driver, electrician) and vigorous OPA (farmer, porter). We combined ‘No job’ and ‘sedentary work’ together and used as a reference group in the analysis. Commuting physical activity (CPA) was defined as daily commuting journey to/from work by bicycle or by bus or walking to/from work or no job. Total score of all physical activities was calculated by combining LPA, OPA and CPA in which each category of CPA was counted as 1 score. Smokers were defined as participants who had smoked at least 100 cigarettes in their life time. The smokers were asked how many cigarettes they consumed per day. Participants were classified into one of four categories according to their smoking intensity: non-smoker, less than 5 cigarettes per day, between 5 and 10 cigarettes per day and more than 10 cigarettes per day. Participants also provided information on the age they started and stopped smoking. The number of pack-years of smoking was derived by using smoking intensity and duration, i.e. number of packs of cigarettes per day multiplied by the number of years of smoking. We asked each participant about monthly alcohol consumption within the recent year. The participants that consumed alcohol on a monthly basis were asked to provide the usual amount of grape wine, rice wine, beer and liquor consumption separately. One drink was defined as one 4-ounce glass of grape wine, 4.8-ounce of rice wine, 12-ounce can of beer or 1 ounce of liquor, all of which contain approximately 0.5 ounce of absolute alcohol [19]. Participants were classified into 4 categories according to their alcohol intake: non-drinkers, light, moderate and heavy drinkers. During the interview participants were asked about familial history of hypertension, hyperlipidemia, diabetes, coronary heart disease (CHD) and stroke of their parents. Familial history of diseases was considered positive if their parents had any of the diseases mentioned above. We also investigated some food intakes such as red meat (including pork, beef and lamb), white meat (including chicken, duck and goose) and fish in the survey. The participants were asked how frequently (daily, weekly, monthly, yearly, or never) they consumed these food groups over the past year, followed by a question on the amount consumed in liang (50 grams/liang) per unit of time. Tea consumption was defined as tea drinking at least three times a week for more than six months continuously. According to standard protocol, anthropometric measurements of weight, height, waist circumferences were taken twice. A third measurement was taken if difference between the first two measurements was larger than 1 cm for height and waist circumference or 1 kilogram (kg) for weight. Two measurements for each participant help prevent reading and typing errors. Intra observation variations of three anthropometric measures are small and averages of coefficient variation are 0.27%, 0.02% and 0.24% for height, weight and waist circumferences, respectively. Therefore we applied an average for two readings for height, weight and waist circumferences in this study. From anthropometric measures, BMI was created by weight in kilograms divided by the square of height in meters. Socio-demographic factors such as age at interview, education (none, elementary school, middle/high school, college and above), family income in yuan/month (≤500, 501–1000, 1001–2000, 2001–3000, 3001–5000, >5000), occupation and medical history of diabetes and CVD were included in the analyses as potential confounders.

### Blood glucose and lipid measurements

At the time of in-person interview, a 10 ml overnight fasting blood sample was drawn into an EDTA vacutainer tube to measure serum lipids and glucose. The fasting time was verified before blood sample collection and participants who had not fasted for at least 8 hours did not have their blood drawn. The samples were kept in a portable Styrofoam box with ice packs (0–4°C) and were shipped to a central CDC laboratory in Nantong. Serum samples were obtained by centrifuge. Glucose and lipid levels were measured within 6 hours of sample separation. Remain specimens were stored at −70°C until other laboratory assays could be conducted. Serum glucose, HDL-cholesterol and triglyceride levels were analyzed enzymatically using reagents from the Shino-test Corporation, Japan. Serum levels of glucose and lipid profile of 20,502 participants were measured by the Nantong CDC using an Automatic Chemistry Analyzer (Hitachi 7180, Tokyo, Japan). Both inter- and intra- assay of variation (coefficient of variation, CV) were less than 3.5% for glucose, triglycerides and HDL-cholesterol.

### Criteria of metabolic syndrome

Criteria of metabolic syndrome according to ATPIII, IDF and ATPIII-modified are presented in Table 1.

**Table 1:** Criteria of metabolic syndrome used in current study.

MS components	ATPIII [3]	IDF [4]	ATPIII-modified [5]
To be diagnosed as MS:	3 or more components	central obesity plus at least two other components	3 or more components
Waist circumference	> = 88 cm	> = 80 cm	> = 80 cm
blood pressure	SBP≥130 or DBP≥85 mmHg or Taking hypertension medication	Same as ATPIII criteria	Same as ATPIII criteria
serum glucose level	> = 6.1 mmol/L or taking diabetes medication	> = 5.6 mmol/L or taking diabetes medication	Same as IDF criteria
serum triglyceride level	>1.70 mmol/L	>1.70 mmol/L or taking abnormal lipid medication	Same as IDF criteria
HDL-cholesterol	<1.29 mmol/L	<1.29 mmol/L	<1.29 mmol/L

### Statistical analysis

We calculated the prevalence of metabolic syndrome according to the three criteria and compared their consistence using the Cohen's Kappa statistic. ANOVA was applied to compare continuous variables and 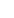 test was used to evaluate proportions of discrete variables between healthy women and MS cases. Non-conditional logistic regression was used to assess associations between metabolic syndrome (dependent variable) with familial history, smoking status, alcohol consumption and physical activity. The regression model was adjusted for age (continuous), BMI (continuous), education, income, marital status, occupation, tea drinking and pre-existing CVD and diabetes. Test for linear trend was performed by entering the ordinal exposure (such as physical activity, smoking (pack-year) and alcohol intake) as continuous parameters in the models. Restricted cubic spline model was used to do curve fitting between ORs of MS and alcohol consumption (continuous variable) and a figure was presented as curve fitting result. All analyses were performed using SAS (version 9.2; SAS Institute, Cary, NC) and all tests of statistical significance were based on two-sided probability.

## Results

A total of 2,974 (22.0%), 2,287(16.9%) and 3,142 (23.3%) participants met MS criteria of the IDF, ATPIII and ATPIII-modified, respectively. The levels of agreement of the ATPIII with IDF and ATPIII-modified criteria were good, with Kappa statistics of 0.75 and 0.80, respectively. The agreement between IDF and ATPIII-modified was almost perfect agreement with kappa = 0.95. A total of 22.9% participants had low HDL-cholesterol (<1.29 mmol/L), while 24.5% had high triglycerides (>1.70 mmol/L) and 33.7% had hypertension (> = 130/85 mmHg and/or taking hypertension medication). There were 884 women (6.6%) with fasting glucose levels of at least 5.6 mmol/L and 613 (4.5%) with fasting glucose levels of 6.1 mmol/L or higher. There were 7,102 women (52.6%) that had a high waist circumference according to ethnic specific cut point (80 cm for Chinese women), among them only 3,176 participants (23.5%) met the criteria for high waist circumference from the ATPIII criteria (88 cm).

Presented in Table 2 are selected demographic and risk factor characteristics of MS for participants in this study. Healthy women and MS cases were similar with respect to marital status, cigarette smoking habits, leisure time exercise and intake of red meat, white meat and fish. MS cases were more likely to be older; have a higher weight, waist circumferences and BMI; more tea consumption; lower education level and lower income per person when compared to healthy women. MS cases were less likely to have alcohol consumption compared to healthy women. The prevalence of diabetes and CVD were higher in MS cases.

**Table 2:** Characteristics of women in rural China by MS cases and health participants in the NMSS during 2007–2008.

	IDF criteria			ATPIII criteria			ATPIII-modified criteria		
	Cases	Non-cases	P	Cases	Non-cases	P	Cases	Non-cases	p
	(n = 2965)	(n = 10540)		(n = 2287)	(n = 11218)		(n = 3160)	(n = 10345)	
Age (years, 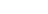 )	57.3±0.17	52.0±0.12	<0.01	57.9±0.19	52.2±0.11	<0.01	57.4±0.17	51.9±0.12	<0.01
Height (cm, 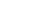 )	156.5±0.11	155.6±0.06	<0.01	156.5±0.12	155.6±0.05	<0.01	156.3±0.10	155.6±0.06	<0.01
Weight (kg, 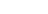 )^1^	66.3±0.17	55.9±0.09	<0.01	67.3±0.19	56.3±0.08	<0.01	65.5±0.16	55.9±0.09	<0.01
Waist (cm, 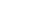 )^1^	90.6±0.16	78.2±0.09	<0.01	91.8±0.19	78.7±0.08	<0.01	89.6±0.16	78.3±0.09	<0.01
BMI ( 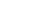 )^1^	27.1±0.06	23.0±0.06	<0.01	27.5±0.07	23.2±0.03	<0.01	26.8±0.06	23.1±0.03	<0.01
Red meat (g/d, 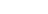 )^1^	27.3±2.0	28.3±1.0	0.67	26.0±2.27	28.5±1.01	0.32	26.9±1.93	28.4±1.06	0.49
White meat (g/d, 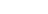 )^1^	17.2±0.46	16.9±0.24	0.61	16.5±0.53	17.1±0.24	0.27	16.9±0.45	17.0±0.25	0.80
Fish (g/d, 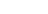 )^1^	29.2±1.20	29.1±0.63	0.96	29.4±1.37	29.0±0.61	0.80	29.1±1.17	29.1±0.64	0.99
Education (%)									
Primary/under	75.5	60.5		77.0	61.1		75.2	60.3	
Middle school	18.3	28.8		16.5	28.6		18.2	29.1	
High/above	6.2	10.7	<0.01	6.5	10.3	<0.01	6.6	10.6	<0.01
Marriage status									
Yes	89.3	90.3		88.2	90.4		89.2	90.3	
No^2^	10.7	9.7	0.12	11.8	9.6	<0.01	10.8	9.7	0.05
Income/person(%)									
≤500 yuan	68.3	64.3		68.8	64.4		68.3	64.2	
501–1000 yuan	25.7	30.1		25.5	29.9		25.7	30.2	
≥1001 yuan	6.0	5.6	<0.01	5.7	5.7	<0.01	6.0	5.6	<0.01
Farmer (%)									
Yes	77.2	66.7		77.7	67.3		76.8	66.7	
No	22.8	33.3	<0.01	22.3	32.7	<0.01	23.2	33.3	<0.01
Ever smoker (%)									
Yes	4.2	3.8		4.5	3.7		4.5	3.7	
No	95.8	96.2	0.31	95.5	96.3	0.11	95.5	96.3	0.05
Ever drinker (%)									
Yes	10.0	11.5		10.1	11.4		10.0	11.5	
No	90.0	88.5	0.02	89.9	88.6	0.09	90.0	88.5	0.02
Tea consumption (%)									
Yes	12.5	10.0		13.4	9.9		12.4	10.0	
No	87.5	90.0	<0.01	86.6	90.1	<0.01	87.6	90.0	<0.01
Exercise (%)									
Yes	9.7	10.0		9.9	9.9		9.7	10.0	
No	90.3	90.0	0.61	90.1	90.1	0.93	90.3	90.0	0.60
Diabetes (%)									
Yes	8.4	0.8		10.9	0.8		8.7	0.6	
No	91.6	99.2	<0.01	89.1	99.2	<0.01	91.3	99.4	<0.01
CVD (%)									
Yes	1.1	0.4		1.3	0.4		1.1	0.4	
No	98.9	99.6	<0.01	98.7	99.6	<0.01	98.9	99.6	<0.01

1 Adjusted for age at interview.

2 including widowed, divorced/separated and unmarried.

Table 3 shows relations of familial history (including hypertension, diabetes, hyperlipidemia, CAD and stroke) and physical activity with MS. Hypertension, diabetes and stroke are three main risk factors of familial history. A woman with familial history of hypertension, diabetes or stroke had 50% higher risk of getting MS, compared with those women without these diseases in their familial history. Furthermore, odds ratio of MS increased in proportion to the number of these diseases in their familial history (P<0.001). We did not find any association between LPA, CPA and the prevalence of MS. However, vigorous-intensity OPA was reduced 24% of MS risk, compared with those women without job or having sedentary job. A combination of LPA, OPA and CPA was associated with decreased risk of MS but this association was slightly attenuated by multiple adjustments.

**Table 3:** Association of family history and physical activities with prevalence of MS according to IDF criteria among 13,505 women in rural China, the NMSS during 2007–2008.

	Percentage of cases (%)	OR(95%CI)^a^	OR(95%CI)^b^
	Familial history		
Hypertension			
No	19.8	1.0	1.0
Yes	27.1	1.61(1.43–1.81)	1.59(1.41–1.80)
Hyperlipidemia			
No	21.3	1.0	1.0
Yes	28.6	1.55(0.93–2.58)	1.50(0.90–2.51)
Diabetes			
No	21.2	1.0	1.0
Yes	29.4	1.72(1.25–2.35)	1.66(1.21–2.28)
CAD			
No	21.3	1.0	1.0
Yes	22.8	1.11(0.85–1.44)	1.10(0.85–1.43)
Stroke			
No	21.1	1.0	1.0
Yes	27.9	1.34(1.04–1.72)	1.34(1.04–1.73)
Risk score			
0	19.6	1.0	1.0
1	26.0	1.53(1.36–1.73)	1.52(1.35–1.71)
≥2	28.1	1.58(1.27–1.97)	1.56(1.25–1.95)
P for trend		<0.001	<0.001
	Physical activity		
LPA^c^			
No	22.2	1.0	1.0
Yes	20.9	1.07(0.95–1.21)	1.05(0.93–1.19)
LPA (categories)			
No	22.2	1.0	1.0
Light-intensity	20.5	1.10(0.94–1.29)	1.06(0.90–1.24)
Moderate-intensity	20.6	1.02(0.86–1.21)	1.02(0.86–1.22)
Vigorous-intensity	27.6	1.23(0.80–1.90)	1.20(0.77–1.88)
P for trend^g^		0.2995	0.4399
OPA^d^			
No or sedentary work	23.4	1.0	1.0
Light-intensity	15.5	0.66(0.53–0.83)	0.70(0.55–0.88)
Moderate-intensity	12.6	0.66(0.53–0.82)	0.72(0.57–0.90)
Vigorous-intensity	24.1	0.68(0.58–0.78)	0.76(0.63–0.91)
P for trend^g^		<0.001	0.0051
CPA^e^			
No work or farmer	24.9	1.0	1.0
By bicycle	15.4	1.03(0.82–1.28)	0.93(0.73–1.18)
Walk only	12.6	0.90(0.73–1.11)	0.86(0.69–1.07)
Walk and take bus	24.1	0.90(0.75–1.07)	0.83(0.68–1.02)
Score of total PA^f^			
0	28.0	1.0	1.0
1	19.5	0.91(0.70–1.18)	0.97(0.74–1.26)
2	17.3	0.81(0.61–1.06)	0.84(0.63–1.11)
3	23.0	0.71(0.58–0.86)	0.80(0.65–1.00)
4	20.3	0.71(0.56–0.89)	0.79(0.62–1.00)
> = 5	19.8	0.68(0.52–0.88)	0.78(0.59–1.03)
P for trend^g^		<0.001	0.0223

a Adjusted for age at interview and BMI.

b Adjusted for age at interview, BMI, education level, income status, occupation, ever smoker, ever drinker and tea consumption in family history calculation, additionally adjusted for history of diabetes and CVD in physical activity calculation.

c LPA: Leisure-time physical activity.

d OPA: Occupational physical activity.

e CPA: Commuting physical activity.

f Score was sum of LPA, OPA, CPA by bicycle, walking and both walking and taking bus.

g P for trend was calculated by entering the ordinal exposure as continuous parameters in the model.

**Figure 1:**
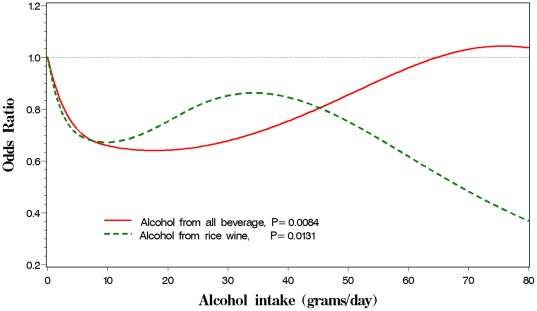
Association of alcohol consumption and MS risk. Legend: Association of metabolic syndrome risk and alcohol intake by all beverage and rice wine among 13,505 women in rural China, the NMSS during 2007–2008.

Table 4 presents the relationship of MS with cigarette smoking and alcohol consumption. Neither smoking intensity (cigarettes smoked per day) nor smoking duration (years of smoking) was associated with the prevalence of MS. However, pack-year of smoking was observed to be associated with the prevalence of MS. A woman smoked more than 20 pack-years had increased 81% of MS risk, compared with non-smokers after adjustment for multiple confounders (P = 0.04 for trend test). A total of 11.2% participants consumed alcohol in our study. Among them, 95.6% reported to be beer or rice wine consumers and 1.8% were grape wine consumers. Thus, we combined rice wine and grape wine together to form a wine consumption group in our study. Consumption of alcohol may decrease 25% of MS risk, compared with non-consumption. Interestingly, drinking liquor or beer was not significantly associated with low prevalence of MS. As a contrast, wine consumption (primarily rice wine) was inversely associated with the prevalence of MS. A woman having more than 12.8 grams of alcohol intake from wine per day was associated with decreasing 34% MS risk. If we combine alcohol intake from beer, liquor and wine together, we observed that alcohol had protective effect on the prevalence of MS (P<0.001) and this protective effect was strongest at about 15–20 g alcohol consumed per day and may gradually disappear if more than 20 g of alcohol was consumed daily. Our observations showed that the more wine consumed, the better protective effect (figure 1). We did not find any interaction effects among risk score of familial history, smoking behavior (pack-year), wine consumption and OPA (data not shown).

**Table 4:** Association of smoking and alcohol consumption and prevalence of MS according to IDF criteria among 13,505 women in rural China, the NMSS during 2007–2008.

	Percentage of cases (%)	OR(95%CI)^a^	OR(95%CI)^b^
Smoking status			
Never	21.9	1.0	1.0
Ever	23.7	1.09(0.85–1.38)	1.15(0.90–1.48)
Smoking rate (cigars per day)			
Never	21.9	1.0	1.0
≤5	20.3	0.85(0.56–1.29)	0.94(0.62–1.44)
≤10	24.2	1.10(0.75–1.61)	1.11(0.74–1.65)
>10	31.2	1.65(0.97–2.79)	1.80(1.03–3.13)
P for trend^c^		0.1785	0.1017
Years of Smoke			
Never	21.9	1.0	1.0
≤30	19.7	0.75(0.49–1.16)	0.76(0.49–1.19)
≤44	25.6	1.24(0.80–1.92)	1.31(0.82–2.08)
>44	27.4	1.42(0.91–2.20)	1.56(0.99–2.46)
P for trend^c^		0.1764	0.0761
Smoking pack-year			
Never	21.9	1.0	1.0
≤7.5	19.9	0.79(0.51–1.22)	0.79(0.51–1.25)
≤20	25.6	1.02(0.65–1.62)	1.12(0.69–1.79)
>20	27.4	1.67(1.09–2.58)	1.81(1.15–2.84)
P for trend^c^		0.1050	0.0451
Ever drink			
Never	22.2	1.0	1.0
Ever	19.7	0.73(0.63–0.85)	0.75(0.64–0.88)
Beer: months of drink			
0	22.1	1.0	1.0
≤3	21.5	0.90(0.74–1.11)	0.95(0.78–1.17)
>3	18.8	0.66(0.47–0.94)	0.69(0.48–0.99)
P for trend^c^		0.0176	0.0671
Alcohol from beer (g/d)			
0	22.1	1.0	1.0
≤3.1	19.7	0.73(0.55–0.95)	0.77(0.58–1.01)
≤6.2	22.1	0.95(0.72–1.25)	1.01(0.76–1.32)
>6.2	20.0	0.85(0.57–1.26)	0.89(0.60–1.33)
P for trend^c^		0.1341	0.3463
Liquor: months of drink			
0	21.9	1.0	1.0
≤5	25.2	1.01(0.71–1.44)	1.05(0.74–1.51)
>5	19.1	0.71(0.48–1.04)	0.70(0.47–1.03)
P for trend		0.1287	0.1421
Alcohol from liquor (g/d)			
0	21.9	1.0	1.0
≤6.2	23.6	0.96(0.64–1.43)	1.01(0.68–1.52)
≤20.5	19.3	0.66(0.39–1.11)	0.66(0.38–1.13)
>20.5	22.5	0.92(0.59–1.43)	0.88(0.55–1.40)
P for trend^c^		0.2561	0.2434
Wine: months of drink			
0	22.3	1.0	1.0
≤6	20.3	0.71(0.56–0.89)	0.73(0.58–0.93)
>6	14.7	0.55(0.40–0.75)	0.56(0.40–0.77)
P for trend^c^		<0.001	<0.001
Alcohol from wine (g/d)			
0	22.3	1.0	1.0
≤4.1	20.3	0.71(0.53–0.98)	0.75(0.55–1.03)
≤12.8	16.8	0.56(0.40–0.78)	0.57(0.40–0.80)
>12.8	16.9	0.64(0.47–0.88)	0.66(0.48–0.91)
P for trend^c^		<0.0001	<0.0001
All sources of alcohol (g/d)			
0	22.2	1.0	1.0
≤5.7	21.0	0.77(0.60–0.99)	0.81(0.63–1.05)
≤17.7	18.9	0.69(0.53–0.89)	0.72(0.56–0.94)
>17.7	19.0	0.73(0.57–0.94)	0.73(0.56–0.95)
P for trend^c^		0.0001	0.0005

a Adjusted for age at interview and BMI.

b Adjusted for age at interview, BMI, education, marital status, income, occupation, ever smoker, ever drinker, tea consumption, history of diabetes and CVD.

c P for trend was calculated by entering the ordinal exposure as continuous parameters in the model.

## Discussion

In this current study, 17% of the women met ATPIII criteria, 22% and 23% of the women met current IDF and ATPIII-modified criteria. Cigarette smoking and family history were associated with increasing risk of MS but occupational physical activity and moderate drinking were associated with a lower risk of MS.

Epidemiological studies have reported that the prevalence of MS varies widely, from 7.1% to 41.6% across different studies, which might partially be due to the use of different definitions for MS or different study populations [3], [20]. Overall, the prevalence of MS in our study was higher than that reported from an earlier study in China. In the study of 27,739 participants (35–64 years) conducted in 11 provinces in 1992, the prevalence of MS in women was 14.2% [20]. In recent years the prevalence of obesity, diabetes, dyslipidaemia, and hypertension has rapidly increased in China due to economic development and its associated changes in lifestyles [21]. As a consequence, MS has been an emerging epidemic in China [22]. Yang et al. found that MS prevalence in women is 23.3% using IDF criteria and 29.1% using modified-ATPIII criteria with the kappa coefficient of 0.80 when he investigated 15,838 Chinese adults aged 35 to 74 years [23]. The prevalence of MS in the elderly in China is higher according to IDF criteria, varying from 25.81 to 34.8% [17]. The prevalence in our study is comparable with the results in these two studies. However, it is still lower than that of some developed countries or areas. Data from a Korean population showed the prevalence of MS for women was 26.6% according to the IDF and 31.9% according to the ATPIII-modified [24]. In a recent study in Taiwan, the prevalence of MS in the IDF and ATPIII-modified was 34.3% and 36.6% [7]. The prevalence of these two studies was higher than in our study. Pan et al. reported that prevalence of MS is 46.1% among US women based on the IDF definition [8], which is higher than in both European populations [1], [25] and in our study population.

Our study showed that the parental history of hypertension, diabetes and stroke was three independent predictors of MS in a large population-based study. Moreover, the odds ratio of MS increased in proportion to the number of these predictors reported in familial history. In agreement with our data, similar findings have been reported in previous studies. A recent prospective cohort study by Mattsson et al. demonstrated that familial history of hypertension and diabetes are two determinants of MS after 21-year follow-up [26]. Another cross-sectional study in Asia reported that MS is associated with familial history with RR = 1.5 (P<0.05) [11]. There may be some underlying genetic variants which predispose some vulnerable individuals to the development of MS [27]. Also genes may modify the impact of obesity and other conventional risk factors [26] and consequently relate with the prevalence of MS. A recent meta-analysis study, which included 18 studies, examined the effect of genetic variants in fat mass and obesity related gene (FTO gene) on MS. They found three SNPs (rs9939609, rs8050136 and rs1421085) are strongly associated with MS and suggested that the FTO gene plays a critical role in leading to MS [28]. Another genome-wide screen study for MS susceptibility loci found that a lipid locus (rs964184) is associated with MS and concluded that genes from lipid metabolism pathways have the key role in the genetic background of MS [29]. Because MS is a complex disorder, it is very likely that individual SNPs may contribute little to the onset and development of MS. Therefore, further genetic studies are needed to clarify the involvement of genetic variants and interaction between genetic variants and environmental factors in MS development.

We did not find an association between the prevalence of MS and smoking duration or smoking density because there are only 3% women who ever smoked. Combining years of smoking and number of cigarette smoked per day together we found pack-year was associated with increases risk of MS. This finding is consistent with findings of several previous studies [11], [12]. Smoking more than 20 cigarettes per day is found to be associated with higher risk of MS in European and Asian populations [12], [30] because smoking could increase the risk of MS via the development of abdominal obesity [30] and insulin resistance [31]. But association between smoking and MS is controversial. Two cross-sectional studies in Portugal and Japan found that there is no association between smoking and MS [13], [32]. Furthermore, a prospective study found a ‘protective’ effect against developing MS of smoking 11 or more cigarettes a day among Turkish women after a mean 5.9-year follow-up. The authors explained that smoking might induce weight loss and contribute to protection against abdominal obesity [33]. Some additional confounders should be considered in this study. Smoking is a risk factor for cancers (such as lung cancer) and many other diseases. These diseases could be confounders in this prospective study because people who had cancers or some other diseases may become thin. Also distributions of familial history of diabetes and hypertension and alcohol consumption between healthy people and MS cases may make biases in relative risk (RR) estimations. Thus the results could be more accuracy if models were additionally adjusted for comorbidity, family history and drinking in RR calculations in this study.

Drinking excessive amounts of alcohol regularly for years is toxic to almost every tissue of our body due to disturbance of a wide variety of metabolic functions and organ damage. However, epidemiological and clinical evidence shows that light-to-moderate drinking is associated with a reduced risk of coronary heart disease, stroke and total mortality in middle-aged and elderly people [34], [35]. Data on association between alcohol consumption and MS are limited and inconsistent [11], [36], [37]. A cross-sectional study of the Third National Health and Nutrition Examination Survey conducted in the USA showed that light to moderate alcohol consumption is associated with lower prevalence of MS [36] and Park et al. reported that this association is observed only in women [14]. But Santos et al. did not find any association between alcohol consumption and MS in both men and women [13]. In our study, we found that neither beer nor liquor consumption were related with the prevalence of MS. But total alcohol beverage consumption may decrease the risk of MS under a J-shaped relationship: Alcohol consumption of less than 20 g/day was inversely associated with the prevalence of MS but this association gradually disappeared if drinking more than 20 g/day. This controversy could be related to the complex mechanistic relation between alcohol consumption and each component of MS. Yoon et al. reported that alcohol consumption is associated with high blood glucose and higher triglycerides in women and concluded that metabolic syndrome is inversely associated with light alcohol consumption (1–15 g/day) in Korean adults [38]. Besides the effect on MS through different components, this effect is probably dictated by a number of competing and confounding influences, such as type of alcohol beverage consumed. Djousse et al. compared the prevalence of MS cross all beverage types and found OR down to 0.32 for wine drinkers only [37]. Consistent with Djousse's study, we found that only wine consumption was related with constantly decreasing risk of MS as well. The mechanics under this protective effect is plausible, especially for rice wine in China. Leighton et al. suggested that the beneficial effect of wine could be related to its content in polyphenols via effect of nitric oxide synthase [39]. Corder et al. found that red wine has been shown to have higher levels of bioflavonoids, which may induce endothelium-dependent dilation of blood vessels and suppress the synthesis of endothelin-1 (ET-1), a peptide that has a vasoconstricting effect [40]. This may partially explain association of wine consumption and lower risk of MS.

Several studies found that physical activity has the beneficial effect on the prevalence of MS [15], [16]. Increase in physical activity improves individual metabolic parameters or combinations of them directly by promoting weight reduction [41], [42], [43]. However, we did not find association between leisure-time physical activity (LPA) and MS in our study. A possible reason is that among all participants only 10% of them took part in LPA with moderate- or vigorous-intensity. In contrast, among participants with moderate- or vigorous-intensity of OPA 80% women did not take LPA. Therefore, the association of LPA with MS is hard to detect if a reference group is composed of those participants who have high levels of occupational physical activity. Although LPA has been associated with a lower risk of MS in some studies as mentioned above, there are very few studies available that have examined associations between MS and occupational physical activity (OPA), especially in the rural areas. A MS study in urban Sweden reported that work-related PA is not associated with MS in men but is associated with MS in women [44]. In a Go Red North Dakota Study, authors reported that no direct relationship between OPA and MS is determined and the odds for MS are similar among women employed in moderately active or heavy work yet completing insufficient LPA compared with women doing sufficient LPA [45]. However, we found that occupational physical activity (OPA) was inversely associated with the prevalence of MS with OR = 0.74 (P = 0.0065). A protective role for OPA in the development of MS is not clear. It could be that the majority of women (78% in our study) take heavy labour (moderate- to vigorous- intensity), which is similar with muscular strength exercise to some extent and therefore, is associated with lower risk of MS [46].

In developing countries, the prevalence of MS tends to increase. High prevalence of MS has been reported from the Philippines (19%), Malaysia (24.2%), India (28.8%), Turkey (33.4%), Iran (33.7%), Venezuela (31.2%) and Brazil (25.4%) [47]. Obesity could be one of the critical causes of high MS prevalence since the prevalence of MS increases simultaneously with the rising prevalence of obesity. A study in 1998 reported that the prevalence of obesity increased from 2.3% to 19.6% within the last ten years in developing counties [48]. Surprisingly, two recent studies found that among rural women the prevalence of MS is 31.25% in Bangladesh and 36.4% in India using ATPIII-modified criteria [49], [50], which are higher than that in our study. The prevalence of MS in developing countries is sparse because of using different criteria in MS diagnosis. In addition, variation in the prevalence of MS could be due to heterogeneity of populations (age distribution, socioeconomic strata or nutritional status) or due to different ethnic populations (different genetic characteristics). In line with our data, several studies from developing countries suggested that physical activity and moderate drinking are protective factors and family history of diabetes, hypertension and CVD and cigarette smoking are risk factors of developing MS [51], [52], [53]. Furthermore, a study among urban Mexican found that both leisure-time and workplace activity at different intensity levels could significantly reduce the risk of MS [54] and this finding was also observed in an animal experiment [55] in Brazil. Sangun et al. reported that in obese children and adolescents MS prevalence is significantly higher in children who have a family history of heart disease, diabetes, obesity and hypertension [56]. Anjana focused on family history of diabetes and found that familial history of type II diabetes increases risk of not only glucose intolerance but also other cardiometabolic risk factors like low high-density lipoprotein cholesterol and high blood pressure in Asian India adolescents [57]. Interestingly, a study in Brazil found that factors of smoking, alcohol consumption and physical activity are not associated with MS in young people [58].

Strengths of this study are a population based study in rural China, extensive information on confounders and a relatively large sample size; all variables were measured using standard methods and vigorous quality control. One limitation of the study is that there are 1675 women who had no information of familial history and they were excluded from familial history analysis in this study. This exclusion could make a bias in OR estimation. Another limitation is about our cross-sectional study design that does not allow us to draw any causal inference. Therefore future prospective studies should be used to confirm the association between exposures in our study and MS. Additionally our study did not include individuals in younger and older age groups, thus it did not provide prevalence estimates among these age groups.

In summary, MS becomes more prevalent among Chinese women regardless of which criterion used to define MS. The prevalence is very low among young women and increases dramatically among middle-aged and elderly women in China. Our data has provided evidence that all alcohol beverage intake (especially rice wine intake) and occupational physical activity are associated with a lower prevalence of MS, but familial history and smoking status are two risk factors of MS among women living in rural China. One of principle implications of our findings is in highlighting the importance of physical activity and moderate drinking for the prevention of MS in rural China and possibly in other developing countries. Another implication is that our findings will help healthcare providers identify those individuals who are at great risk of developing MS and focus more efforts on this special population in rural China.
